# “I Didn’t Reveal My ART Status Because I Didn’t Have Money to Fetch the Transfer Letter”– Understanding Lack of Treatment Disclosure at Presentation to Care in South Africa: A Qualitative Study

**DOI:** 10.1007/s10461-024-04553-2

**Published:** 2024-11-25

**Authors:** Nsika Sithole, Busisiwe Nkosi, Janet Seeley, Ruanne V. Barnabas, Mark J. Siedner, Mosa Moshabela

**Affiliations:** 1 Africa Health Research Institute, Somkhele Campus, R618 Enroute to Hlabisa, Mtubatuba, KwaZulu-Natal, 3935 South Africa; 2 University of Toledo, Toledo, OH USA; 3 London School of Hygiene and Tropical Medicine, London, UK; 4 Division of Infectious Diseases, Massachusetts General Hospital, Boston, MA USA; 5 Harvard Medical School, Boston, MA USA; 6 University of KwaZulu-Natal, Durban, South Africa

**Keywords:** HIV, ART status, Disclosure, Clinic transfers, South Africa

## Abstract

We explored why people may not reveal their antiretroviral therapy (ART) status when presenting for HIV care, and how a linked electronic system may help address this phenomenon. Data were collected from March to October 2023 from people who presented to clinics for an HIV test in KwaZulu-Natal, South Africa but found to be on ART, as well from staff at those clinics. Themes drawn from the Andersen’s health service utilization framework and the domains of a HIV self-management framework were used to guide the analysis. Twenty five people participated in the interviews (18 individuals on ART and seven clinic staff). People did not reveal ART use due to fear of being reprimanded by clinic staff for interrupting treatment or missing clinic visits, with the main reason being administrative challenges, such as requiring a transfer letter to be allowed to access treatment at a new clinic. Some felt ashamed about actions such as buying ART from the black market due to challenges finding treatment. Others wished to present to new clinics because of employment changes, overcrowding in present clinics, missing clinic visits and experiencing stigma. Participants supported the use of a linked electronic system because all medical records would be accessible to health workers in all facilities. People in HIV care in our study demonstrated resilience, finding creative solutions by moving to new clinics to access ART. A linked electronic system which combines records at all health facilities in KwaZulu-Natal could enable individuals to access treatment from any clinic.

**Supplementary Information:**

The online version contains supplementary material available at 10.1007/s10461-024-04553-2.

## Introduction

Despite antiretroviral therapy (ART) being freely available for over 10 years, HIV mortality persists in South Africa [1, 2]. Many people living with HIV (PWH) face unique challenges that require them to balance their mobility seeking employment with the need to sustain access to long term ART for their health [3, 4], increasing susceptibility to treatment interruptions [5]. Even with the introduction of patient centered initiatives such as the Centralised Chronic Medication Dispensing and Distribution (CCMDD) programme which allows PWH to collect ART in the community at pick up points or utilising community health workers or smart lockers, challenges remain [6]. Poverty, migration, the need to prioritize employment over healthcare access, and other structural barriers to care can make sustained engagement difficult, resulting in high turnover, defaulting from care, and clinic switching [5, 7–10].

Yet, transferring between clinics for work or migration can be a major challenge for continuity of care. Patient movement between clinics typically requires an official transfer letter. When this is absent, patients are typically considered lost to follow up (LTFU) from their original clinics [11–14]. LTFU is particularly problematic in Africa where there are limited resources to track and retain patients who have dropped out of HIV care [3]. Indeed, when those who are LTFU are tracked and identified, the most frequent reason for being LTFU was moving to a new area because of a job loss or a change of employment, and most of those identified as LFTU have been found to be engaged in care elsewhere [3].

Despite the growing recognition of self-referral and unregistered clinic switching, our understanding of why it occurs remains poor. A better understanding of the phenomenon and its determinants will be crucial for developing improved strategies to support patient care across geographic regions and in the context of migration and high unemployment. In the Western Cape Province, where integrated data systems allow monitoring of patients across the region, unregistered clinic switching is probably less frequent [15]. However, whether and how this system is acceptable to patients and providers in other regions of South Africa is less well studied.

We examined patient and healthcare worker perspectives on accessing HIV care without disclosing treatment status in rural South Africa. To do so, we conducted a qualitative study among individuals who were discovered to already be on ART at the time of enrolment into HIV care at two clinics in uMkhanyakude District, KwaZulu-Natal (KZN). In addition, we interviewed health workers who provide HIV care at the two clinics and also investigated people’s perspectives regarding the potential of a networked electronic system to address this phenomenon.

## Methods

### Theoretical Frameworks

**Fig. 1:**
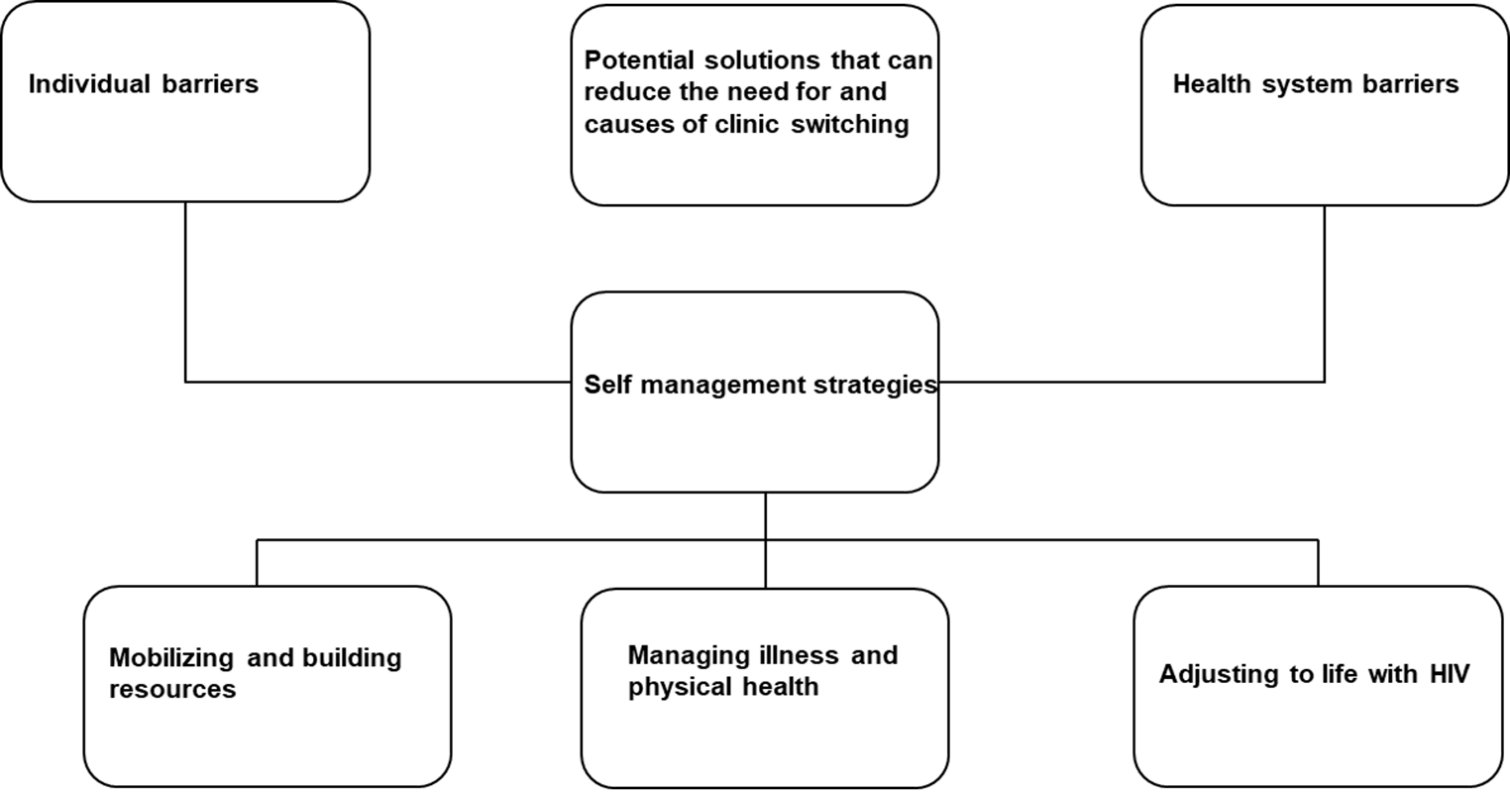
Andersen‘s health utilization model and self management strategies

This study utilised Andersen’s Behavioural Model of health services utilization [16] and the broad domains of a self-management analytical framework developed by Russell et al. [17] to help guide the themes after engaging with the data. The behavioural model is multileveled and incorporates both individual and contextual determinants of health services utilization. The model is built upon the predictors of health services utilization namely (1) predisposing factors, e.g. individual factors such attitudes and beliefs, (2) enabling factors e.g. the distribution of the health service facilities and (3) needs e.g. a perceived change in the individual’s health status [16]. In the context of finding the reasons behind non- disclosure of ART exposure based on Andersen’s Behavioural Model, we changed the predictors of health utilization into the barriers to disclosure and what needs to be done to enhance a people centred approach at the clinics. We also added three domains of self-management which we considered relevant to individuals seeking care outside the standard healthcare system pathways: mobilizing and building resources, managing illness and physical health, and adjusting to life with HIV on ART [17] (Fig. 1).

#### Participant Selection

**Fig. 2:**
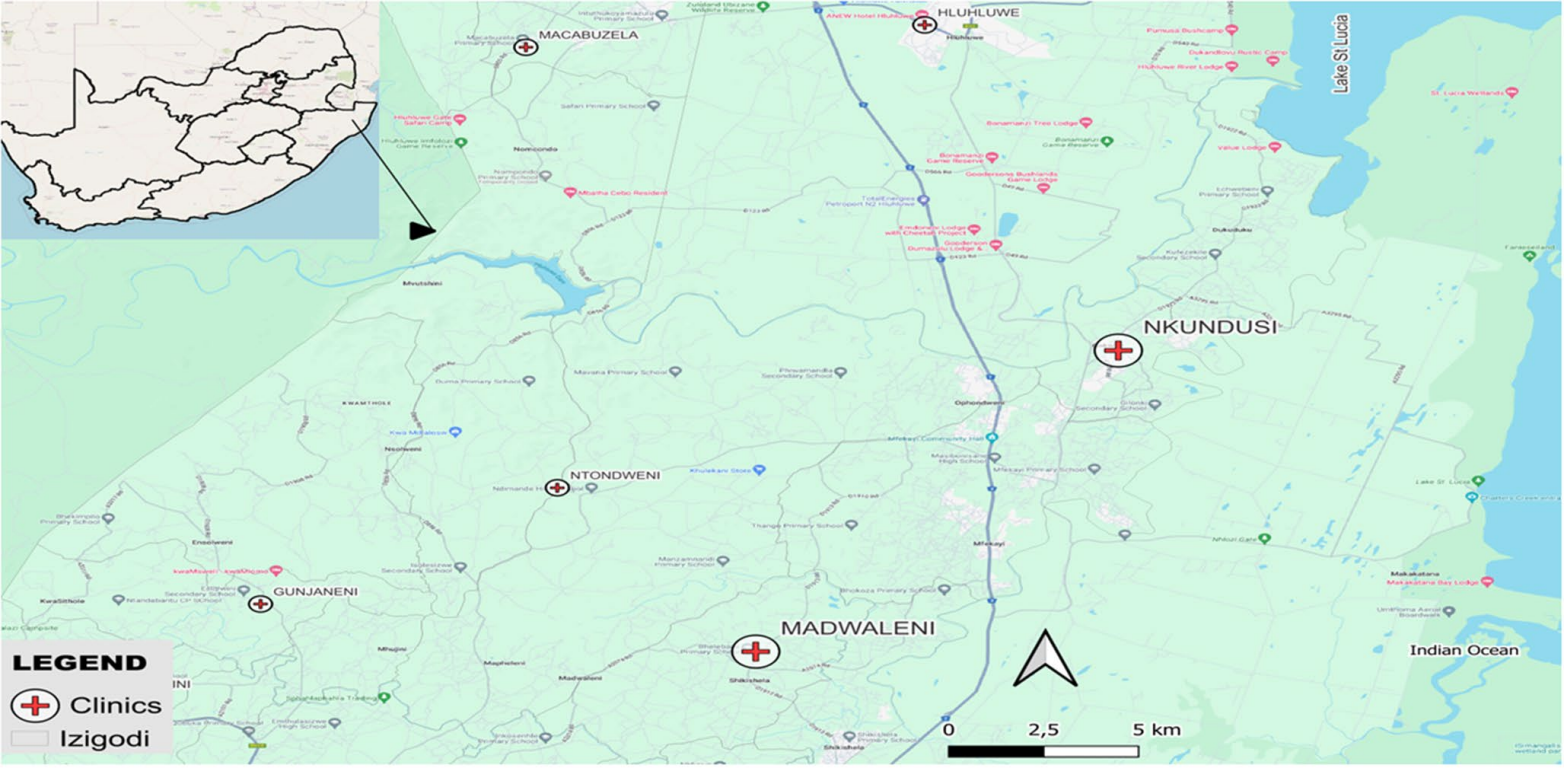
Nkundusi and Madwaleni clinics in the uMkhanyakude district, KwaZulu-Natal, South Africa

We purposively selected a sample of 18 participants from the delivery optimisation of antiretroviral therapy (DO ART) trial’s database. The DO ART trial recruited ART naïve participants or those who interrupted treatment at HIV clinics and the community in KZN [18]. Those selected included individuals who: (1) were screened for the DO ART study at Madwaleni and Nkundusi clinics in the uMkhanyakude district in rural KZN (Figs. 2), (2) tested positive for HIV, (3) requested ART initiation, (4) were found to have an undetectable viral load, (5) had evidence of ART use by pharmacologic testing, and (6) agreed to be contacted in the future by study staff as part of the initial DO ART study consent process. We also purposely selected 4 health workers (2 from each clinic) who are providing HIV care. Finally, to get perspectives on the idea of an electronic data system which is linked to every health facility, we selected 3 Africa Health Research Institute (AHRI) data management personnel to interview.

### Data Collection

Qualitative data collection was conducted from March to October 2023. We reached out to former DO ART trial participants who had consented to being contacted in the future for additional research using contact details stored in the DO ART database. For those whose numbers were not working, we went to the clinics to get the up to date information from the clinic files before making contact. Some participants were found to have relocated to areas outside of the study area. Telephonic interviews were therefore conducted for those who had moved. The lead researcher underwent training in qualitative methods before conducting interviews. He was assisted by 2 research assistants (RAs) with extensive experience in qualitative data collection. All interviews were conducted in isiZulu or English using a semi structured interview guide at a venue which was convenient and acceptable to the participants. The interview guides were structured for specific participants, namely (1) DO ART participants (Appendix), (2) health workers providing HIV care at the 2 clinics and (3) AHRI data management staff. The interviews were digitally recorded and transcribed by translators proficient in English. The transcripts were quality controlled by the researcher and a senior RA. To include additional observations that might not have been included in the interview recordings, the researcher and RAs prepared interview summaries on the day of the interviews.

### Analysis

The interview summaries were quality checked against the transcripts and translations by the researcher and senior RA. Primary coding was used to group related content and themes were identified following code categorization using Nvivo software. The codes emerged after an inductive process that involved consistent comparing between the lead researcher and senior RA. Further analysis of emerging themes was guided by both the Andersen’s Behavioural Model of health services utilization and domains of self-management. Discussion in team meetings, which included the researcher, two RAs and a senior social scientist also helped to validate the themes, with the final version verified by other senior researchers on the team.

## Results

**Fig. 3:**
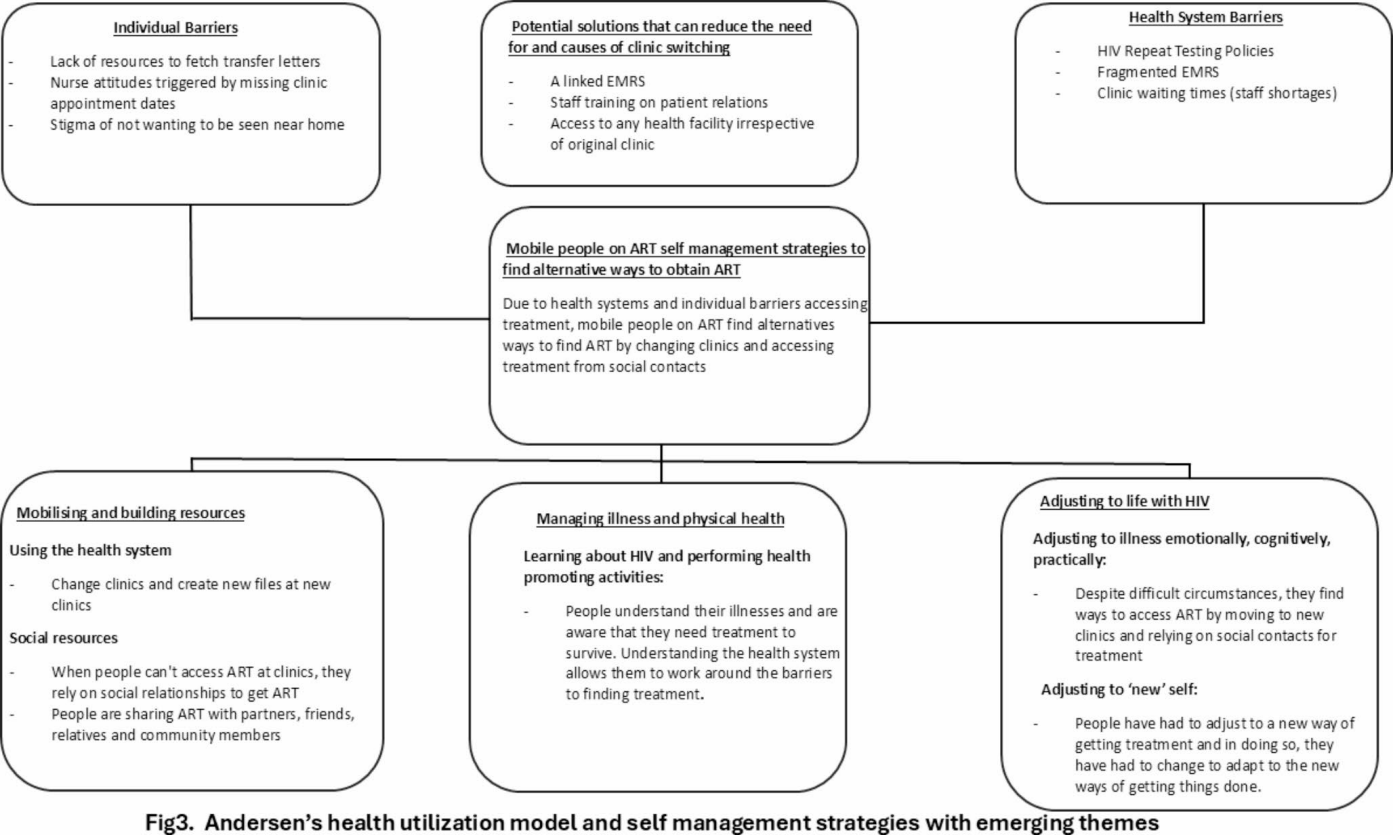
Andersen‘s health utilization model and self management strategies with emerging themes

Twenty-five people participated in the interviews (18 individuals previously exposed to antiretrovirals [13 females/ 5 males aged 24–51 years] (Table 1), 4 nurses, and 3 AHRI data management staff). Emerging themes were grouped guided by the theoretical frameworks after engaging with the data (Fig. 3).

**Table 1:** Participant characteristics and number of stated ART collection clinics from individuals who were discovered to already be on ART at the time of enrolment into HIV care at two clinics in uMkhanyakude District, KwaZulu-Natal

Study ID	Gender	Age	ART collection clinics	Original clinic
Participant 01	M	37	01	Nkundusi
Participant 02	F	42	02	Madwaleni
Participant 03	F	24	02	Nkundusi
Participant 04	F	29	01	Nkundusi
Participant 05	F	24	02	Madwaleni
Participant 06	F	51	02	Madwaleni
Participant 07	M	38	01	Nkundusi
Participant 08	M	32	02	Nkundusi
Participant 09	F	27	02	Nkundusi
Participant 10	M	47	03	Nkundusi
Participant 11	F	34	02	Madwaleni
Participant 12	M	29	01	Nkundusi
Participant 13	F	35	02	Nkundusi
Participant 14	F	37	03	Nkundusi
Participant 15	F	53	02	Madwaleni
Participant 16	F	39	03	Nkundusi
Participant 17	F	48	01	Nkundusi
Participant 18	F	25	02	Nkundusi

### Individual Barriers

People gave different reasons for why they do not reveal their ART histories to health workers at the clinics, including a lack of resources, nurse attitudes and stigma.

### Lack of Resources

Most participants reported a lack of funds to go and fetch transfer letters from previous clinics, with some former facilities being up to 500 km away with costs amounting to ± US $37.

“*I knew that they were going to say I must fetch the transfer letter*, *I did not have money to go there and come back*, *and I needed the treatment”–* Participant 14, female aged 37.

“*The problem is that some are taking treatment in Gauteng*, *because previously there was corona and some of them were retrenched*, *so now they are unable to go back to get transfer letters*”- Nurse 01, male.

### Nurse Attitudes Triggered by Missed Clinic Appointment Dates/Defaulting

Some participants reported negative or accusatory nurse attitudes, which were triggered by missing clinic appointment dates as the reason for people changing clinics and acting as new clients:

“*There is that issue that I missed the scheduled treatment collection date so now*, *the nurse will ask me*, *why I did not come*, *if I miss the date and I come on my date*, *surely I should continue to be given treatment*, *as long as I come to the clinic. The nurse will now clap their hands*, *calling other nurses and say things like*, *“look at a person who does not want to live*, *have a look at a person who does not take care of themselves”*, *it’s not that I am not taking care of myself. We talk nicely with low voices to them*, *but they are shouting for other peoples’ attention*, *that is inhumane*”– Participant 15, female aged 53.

Another participant who experienced problems with the clinic appointment date stated:

“*The other reason is that we are at times not catered for when we’re at the clinics. You find that I missed the date to be at the clinic on the 12th*, *if I come the next day*, *the health workers will not attend to me and say “your date was the 12th so now*, *you are going to wait for us to finish the patients who are booked on this date and you will be the last to be assisted”. This ends up putting a lot of pressure on me to keep up with the appointment date*, *but you find that I did not miss the date on purpose”*- Participant 07, male aged 38.

Furthermore, the difficulty that clients may experience with health care workers was recognised as a problem by some health care workers themselves:

“*And the other thing is the attitude of health care workers*, *because if the patient is a defaulter*, *they think that the nurses will ask them why they defaulted treatment*, *so they opt to pretend as if they are new patients*, *and know that most health workers change clinics all the time and no one will recognise them*, *so they will decide to pretend as if they are new*”- Nurse 02, female.

### Stigma

A nurse highlighted stigma as a possible reason for people to change clinics because they do not want to be seen where they are known.

“*Yes*, *the biggest problem is the transfer letter*, *number 2*, *there is stigma maybe if he/she did not disclose to their new partner*, *I am making an example*”- Nurse 02, female.

### Health Systems Barriers

The following factors emerged and were grouped under the health systems barriers framework: HIV testing policies, fragmented electronic medical records systems (EMRS) and clinic waiting times.

### HIV Testing Policies

Repeating an HIV test while on ART is discouraged in the South African HIV testing guidelines but a few participants, including a nurse, stated that people acted as new clients and retook the HIV test because they wanted to verify a previous test result:

“*She responded by telling me that she thought that the results would be different*, *she was curious to see what the results would be*.” - Nurse 02, female.

“*I just wanted to re-start and test again to see if I will still test positive again*, *that was my aim*.”– Participant 02, female aged 42.

### Fragmented Electronic Medical Record System (EMRS)

Regarding the fragmented EMRS theme, data management personnel stated that the issue of people not revealing their ART status at clinics is caused by the system being unique to each facility, as highlighted in these statements:

“*The problem we have in KZN is that we’ve got electronic information health systems which are siloed*, *meaning that they do not talk to one another. If we were to link systems like NHLS*, *Tier and HPRS*, *we would be able to track how a person interacted with the health system. And this could lead to linking of records between facilities*, *so you’ll be able to see what has happened in the next clinic and so forth. That would solve the problem of people not declaring that they are on ART*”– Data management 02, male.“*So currently the system that records ART treatment in KZN is Tier.net. By contrast in the Western Cape*, *they have an integrated system so irrespective of where the patient is seen*, *the clinician has access to the complete record*, *but in an environment where the default system is still Tier.net*, *you have a problem because there is no mechanism by which if someone has records at a different clinic on Tier.net*, *those records can be seen at different facilities*”– Data management 03, male.

A nurse brought up the important issue of the HIV registry (Tier.net) [19] requiring information which is on a transfer letter to enable the successful transfer of a patient coming from a different clinic. This provides context as to why health workers ask those without letters to fetch them from their previous clinics. The nurse stated the following:

*“Tier.net does not accept a patient without information which is on a transfer letter”-* Nurse 02, female.

### Clinic Waiting Times

Another theme which came up under health systems was the issue of clinic waiting times. A few participants stated that people tend to want to change clinics and go places where they receive attention quicker. They gave the following statements:

“*You will come to the clinic in the morning at 8am*, *and only leave at 2pm*. *These are the reasons people want to change clinics at times*”- Participant 01, male aged 37.“*We are in a queue for a long time*, *approximately 4–5 hours*, *people tend to want to go where they receive care faster”*- Participant 14, female aged 37.

### Domains of Self Management Framework

#### Using the Healthcare System

When the health system appears to be working against patients seeking treatment, they self-manage their conditions by using other available resources and find alternative clinics and sources where they can access treatment. They understand how to work around the system to get treatment. This is highlighted by the following statement:

*“I went to a new clinic and acted as if I’m a new client. To avoid detection*, *I changed my name and gave a fake name so that I could get treatment”.”*- Participant 14, female aged 37.

### Social Resources

Another self-management skill used by people to find treatment in the community is utilising available social resources. They do this by sharing pills with each other when they run out. This highlighted by the following statement:

“*People share pills with one another in the community*, *they sometimes ask them from me*, *and I share with them if I have extra. Most of those who ask are defaulters*” - Participant 05, female aged 24.

Others described sharing treatment with neighbours:

“*When I am out of treatment and cannot go to the clinic*, *I ask my neighbour to share pills with me*”- Participant 06, female aged 51.

### Managing the Illness and Physical Health

People understand about their illnesses and know that they need to take treatment to survive. By understanding the health system, they know how to work around the barriers of finding treatment. This highlights the learning about HIV and performing health promoting activities theme. A nurse highlights this with the following statement:

“*To avoid detection*, *they change their ID numbers*, *they mention a different birth date or slightly different surname in order to get treatment*”– Nurse 02, female.

### Adjusting to Illness Emotionally, Cognitively, Practically

People have had to adjust emotionally, cognitively and practically to the illness in order for them to be able to self manage their conditions. This is shown by this statement:

“*I am facing a challenge because they don’t want to accept me at the new clinic where I’ve relocated*, *and I also don’t have a transfer letter. I have therefore found alternative ways to find treatment*”– Participant 10, male aged 47.

### Adjusting to New Self

People have had to adjust to a new way of getting treatment and in doing so, they have had to change to adapt to the new ways of getting things done. This has caused them to change into a “new self” because they have had to adapt from seeking treatment at clinics to seeking treatment on the black market. This is highlighted by the adjusting to new self theme as shown by the following statement:

“*My partner also shares her pills with me*, *especially during the days when I am waiting for my [black market] supplier (usually 7–10 days)*”– Participant 10, male aged 47.

### Accessing ART from Secondary Sources

A few participants indicated that there are people in the communities who are selling antiretrovirals. Where the sellers were getting their supplies from remained unclear. This is highlighted by the following statements:

“*Sometimes we sit and talk*, *giving each other advice about certain things*, *someone ends up mentioning that they are also on treatment and that is why they are giving me this type of advice because he was also in a similar situation and he does not like other people experiencing the same problems he went through*, *so he gives me treatment and I do pay because I am the one who asked for assistance*, *he asks me to thank him with whatever I can afford*, *and I pay between one hundred and 150 rands*”- Participant 10,male aged 47.“*Someone is selling treatment within the community. Each pill packet costs R50*”- Participant 05, female aged 24.

#### Potential Solutions that Can Reduce the Need for and Causes of Clinic Switching

The needs framework was categorized by what should be done to make the clinics more user friendly so that mobile people on ART can be accommodated. The participants listed the following suggestions: a linked system, staff relations training, permit access to any facilities and patient education.

### Linked Electronic System

A nurse stated the following when asked about the advantages of a linked system:

“*The system will work to our advantage because it will reduce the defaulter rate*, *it will reduce it in a massive way because we will be able to see that this person does not take treatment that side. It will also help us identify misconduct and it will help us manage our stock*”- Nurse 01, male.

However, a few participants were not happy about the idea of a linked system, with a 51 year old female (participant 06) saying the system “*will limit my freedom to go to whichever clinic I like”*. Another commented:

*“I am not happy because if I go another clinic*, *they will see that I am from a different clinic and will ask me why I have come to this clinic. I do not like that”-* Participant 09, female aged 27.

#### Staff Relations Training

A need for staff training was noted by both health workers and patients:

“*That’s where the problem starts*, *when I go to the clinic to collect treatment*, *I need to be patient because the nurses are impatient at times. Our nurses do not have time for patients*”- Participant 01, male aged 37.

A nurse was asked about what needs to be done to improve patient/staff relations and she stated the following:

“*We are attending workshops*, *and they tell us that if a patient has defaulted or is lost to follow up*, *when they come back*, *we need to welcome them in a good manner*, *thank them for coming back and provide counselling which highlights the disadvantages of their deeds and discuss a way forward*”– Nurse 02, female.

#### Permit Access of People with HIV to Any Public Health Facility

A nurse also suggested that the KZN health department should adapt the same policy as other government departments where people are allowed to go to any facility irrespective of where the facility is situated. She stated the following:

“*If I can walk into any home affairs department and get help*, *irrespective of where I am in the country*, *why can’t the department of health do the same where anyone can access care at any health facility*, *even without a physical transfer letter*”– Nurse 02, female.

#### Patient Education

A nurse recommended reinstating pre-treatment adherence classes, previously offered, to educate patients about ART exposure and the impact of adherence to medication for future treatment options, so they were aware of treatment benefits, potential challenges, and coping strategies.

In summary, our results show that mobile people on ART face significant barriers to disclosing their existing ART use at clinics, stemming from both health system and individual factors. Specifically, health system barriers such as administrative and financial challenges, including the requirement for transfer letters to access treatment at new clinics, hindered disclosure. Additionally, individual barriers like fear of encountering scolding by health workers for missing clinic appointments contributed to concealment of ART use. In response to these barriers, mobile people on ART have developed resourceful self-management strategies to access treatment in different ways.

## Discussion

In this study, we sought to understand the reason people do not reveal that they are on ART at treatment initiation at public health clinics in KZN South Africa. We also wanted to get perspectives on a linked EMRS as a mitigation strategy to stop the recurrence of the phenomenon. We identified several factors that lead to individuals on ART not wanting to reveal their ART status to health workers at initiation at clinics.

This study’s findings highlighted administrative and logistical hurdles linked to transferring from one health facility to another. Such barriers impact the effective monitoring of the HIV programme which depends on complete, accurate and timely flow of data between health facilities [20]. Most participants reported lacking funds for transport to fetch transfer letters from previous clinics, as requested by health workers after they disclosed their prior ART treatment at a different clinic. Most of these individuals were labourers who had to relocate for employment opportunities. They often did not have sufficient time to go to prior clinics and request a transfer letter before moving to the next area. This was one of the main reasons why they preferred not to reveal their ART histories to health workers and acted as if they are newly diagnosed, so that they can get treatment.

Health workers also acknowledged the issue of the transfer letter as being at the centre of why people hide their ART histories when initiating. The Tier.net system requires information from a transfer letter to facilitate patient transfers, prompting nurses to request patients to obtain these letters from previous clinics [19, 21]. There were strategies that the nurses acknowledged that they tried using to help patients, like calling previous clinics and asking them to send the letters on email, fax or by Whatsapp, but these also came with barriers like finding clinic telephone numbers not working. A nurse further advocated for the scrapping of the transfer letter if possible, so that patients can utilise any health facility that is most convenient to them. A few AHRI data management staff acknowledged that the issue of people attending multiple clinics in KZN is caused by Tier.net being unique to each health facility, which allows health workers to only see records from the facility they are working from.

A potential solution identified in this study was a linked EMRS. Perhaps if the KZN Department of Health adapted an integrated EMRS that is linked to all facilities like the one used in the Western Cape [15], the issue of duplicated ART prescriptions at public health clinics in KZN can be reduced as this has resource and HIV programmatic data implications [22]. With over 5 million people on treatment, South Africa is home to the world’s largest ART programme [23], so an accurate way to monitor and evaluate treatment programs is needed to evaluate HIV control efforts in the region [24–26]. We recommend future research that compares ART prescription duplications at health facilities in the Western Cape and KZN to see how effective an integrated system can be at reducing multiple clinic visitations for ART prescriptions.

The idea of a linked EMRS was well received by most participants with some highlighting that it has the potential to stop duplicate ART prescriptions. That said, there were a few participants who were not happy about the idea, with one highlighting that their freedom to attend any clinic will be limited. Another negative comment was on a lack of confidence in health workers not divulging patient information to unauthorized people since all patient medical information would be readily available on an integrated EMRS. This finding is consistent with a study conducted in Greece to find the public’s and health worker’s perspectives on an EMRS, which revealed that the majority of the public generally agreed that they would worry about the possibility that a non-authorized third party might gain access to their personal health information, and that they would worry about future discriminations due to possible disclosure of their health information [27]. A data management respondent went as far as saying that once an integrated system was in place, then all health workers working with patient data should sign a non-disclosure agreement to prevent them from divulging information to unauthorized individuals. To guarantee safe and reliable exchange of sensitive health information, the deployment of EMRS must place a high priority on strong privacy and confidentiality controls, clear data sharing protocols, and patient-centred consent mechanisms [28].

A related solution proposed was allowing people to attend a health facility of their choice. An integrated EMRS could promote this practice by allowing patients to be electronically transferred from one clinic to another. In such a scenario, a patient should be able to walk into any clinic in KZN and tell health workers that they are receiving ART from another facility, but they have moved to the area and would like to continue receiving care from that facility while they were there. The health worker would be able to check this information online before transferring the patient, ensuring that the patient’s transition from clinic A to clinic B is documented. This approach would reduce the need for people to attend multiple clinics to find treatment, as nobody would be asked to go and fetch transfers from their original clinics. While this system is not in place, we recommend that health workers working in health facilities in KZN apply a people-centred approach and try to work with mobile ART clients to work around the barriers to obtaining treatment. This will include working on issues such as improving staff relations by educating the staff to be user-friendly at all times and not shout at patients for defaulting or missing clinic appointment dates. It must be acknowledged that a nurse did indicate during an interview that they were attending workshops on how to improve staff and patient relations and we recommend that the KZN department of health extend these workshops to all staff working in all facilities, if this has not already been implemented.

Other factors which contributed to non-disclosure of ART histories included stigma, clinic waiting times, verifying a previous test result, and being denied treatment because of not having transfer letters leading to buying medications on the black market. Some of these factors are consistent with reasons given for transferring care from one health facility to another among HIV clients who were attending HIV clinics in Kenya [8], as well as those given by PWH who disengaged but later resumed care at a health facility in KZN [9]. During an interview with one of the participants who admitted to buying ART on the black market, he explained how he got acquainted with his supplier, and that was through a conversation he had with him about his struggles to get ART after relocating through work without a transfer letter. Future studies should explore the practice and frequency of duplicated ART prescriptions at clinics to better understand this issue, its cause, and potential solutions.

Although these mobile individuals on ART were not following the conventional cascade of care, but they must be recognised for self-managing their situations and working around the barriers they encountered to find treatment. This illustrates that they have adapted to living life with HIV can adopt innovative ways to find treatment and obtain optimal health.

This study had some limitations including that we interviewed participants from two clinics in northern KZN. We recognise that generalizing conclusions from these results might not give a true reflection on other people’s perspectives who are attending other clinics in the province. A strength of this work is that this is the first study, to the best of our knowledge, to report on the reasons why people are not revealing that they are on ART at initiation at public health clinics in South Africa. We hope that the findings of this research may influence a change in policy where mobile people on ART may receive care irrespective of which health facility they attend in KZN.

## Electronic Supplementary Material

Below is the link to the electronic supplementary material.

Supplementary Material 1
