# Supplementary material for: “I Didn’t Reveal My ART Status Because I Didn’t Have Money to Fetch the Transfer Letter”– Understanding Lack of Treatment Disclosure at Presentation to Care in South Africa: A Qualitative Study
Source: AIDS Behav. 2024 Nov 25;29(2):715–24. doi: 10.1007/s10461-024-04553-2 (PMC11813989; doi:10.1007/s10461-024-04553-2)
Supplement: Supplementary file 1 — Supplementary Material 1 [file 10461_2024_4553_MOESM1_ESM.pdf]

Interview questions for former DO ART screening participants:

The following questions will be group into 3 sections, namely:

a) Experience and disclosure
b) Community and stigma
c) Ethics on health workers accessing patient medical records

a) Experience and disclosure

1. One of the topics we are investigating in this study is disclosure of ART or HIV status. Is disclosure of your HIV status and the fact that you are taking ARVs something that you have discussed with your close relatives or friends, partner, or health workers at the clinic?

a. (probe as a follow up to Q1). Could you give us the reasons why you do or do not discuss your use of antiretroviral therapy with friends, family, and healthcare workers?

2. Which clinic are you attending?

a. Are you collecting your ART from this clinic?

b. Is there another clinic that you collect ART from?

c. Is there another clinic that you use to collect treatment for other illnesses?

3. Can you tell me about a time when you did not disclose to a healthcare provider that you were on HIV treatment?

a. (probe) what were the circumstances that led you to do that?
b. What are the advantages of telling your healthcare provider or clinic about treatments you are currently taking?
c. What are the disadvantages of telling your healthcare provider or clinic about treatments you are currently taking?

4. What are your experiences of getting antiretroviral therapy at clinics?

a. What has gone well?
b. What has not gone well?
c. Have you ever had an experience where you decided to leave a clinic and go to a different clinic? Why? Tell me about it?

5. What recommendations would you suggest to meet patients' needs to remain engaged and receive antiretroviral therapy consistently at the clinics?

6. Generally speaking, what are reasons that make people with HIV change clinics or healthcare providers?

7. Have you or someone you know ever changed the place where you receive your ART from? Tell me why this was done? Can you give specific examples?

8. Have you had an experience where you have gone to a new healthcare provider for HIV care, but you did not tell the new provider that you are on treatment? Can you tell me about this experience? What were the circumstances that lead you to do so?

b) Community and stigma

9. In your community, is there stigma surrounding HIV? Are people are hiding that they are on ART eg. They don't want the neighbors to know? Or is the community comfortable and everyone is open about HIV and taking ART?

10. Do people on ART in your community ask fellow ART users to collect their treatment at the clinics on their behalf if they cannot collect their treatment themselves?

11. Do people on ART in your community share their treatment with other ART users such as friends, neighbors, or partners if maybe they run out?

12. Have you ever shared your treatment/ someone else shared their treatment with you?

a) Probe, what were the circumstances surrounding this?
b) How did you ensure that you were left with enough treatment to last the period that was given at the clinic?

13. What are other ways that you or others use to obtain antiretroviral therapy for both you and your loved ones?

c) Ethics on health workers accessing medical records

14. What is your opinion on health workers sharing/accessing patient health information between clinics or within the healthcare system to better understand patient medical and treatment history?

a) What are the advantages of healthcare workers knowing about your health and prior treatments?
b) What are the disadvantages?
c) How would you feel about a system that allows for the electronic sharing of your medical records across all clinics in the area, so that healthcare workers at a new clinic can instantly access your medical history, current medications, and previous care, ensuring that your health information follows you wherever you go?
d) Would you consent to have your health records from other health facilities shared/accessed by health workers?
e) Would you have concerns regarding your right to refuse such a procedure? If so, probe, what are the concerns?
f) Would you have concerns regarding the privacy / confidentiality of your medical records? If so, probe, what are the concerns?
